# Supplementary material for: A Novel Approach for Glioblastoma Treatment by Combining Apoptosis Inducers (TMZ, MTX, and Cytarabine) with E.V.A. (Eltanexor, Venetoclax, and A1210477) Inhibiting XPO1, Bcl-2, and Mcl-1
Source: Cells. 2024 Apr 4;13(7):632. doi: 10.3390/cells13070632 (PMC11011525; doi:10.3390/cells13070632)
Supplement: Supplementary file 1 [file cells-13-00632-s001.zip › cells-2905175-supplementary.pdf]

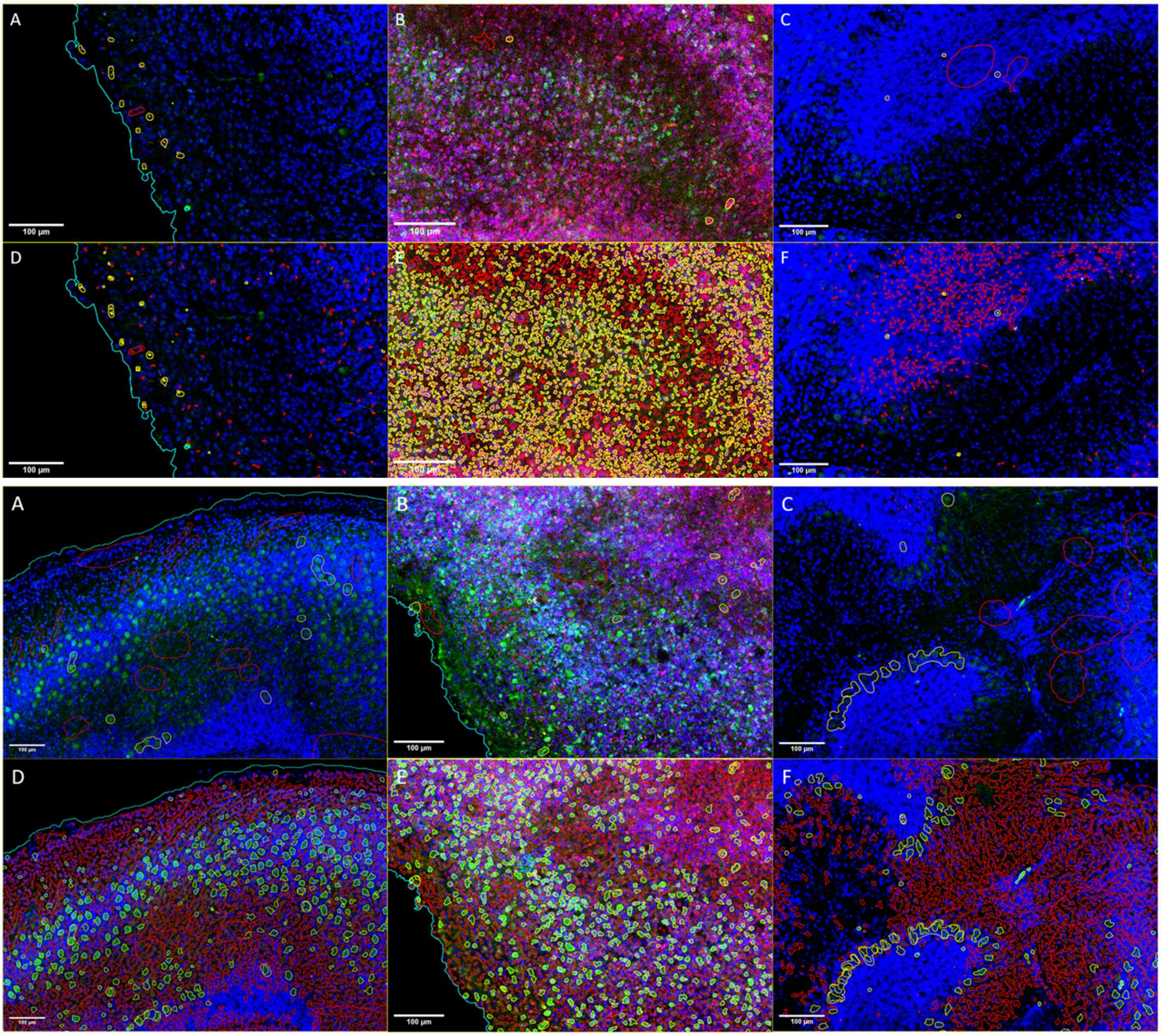

Figure S1. Training of Classifiers for PI (top panel) and Caspase 3 (bottom panel) exemplified by images from the groups „Control“ (DMSO, left), „Staurosporin“ (middle), and „TMZ“ (right). In figures A – C, only the annotations to train the classifiers are shown, whereas in figures D - F the detections obtained in the first step of image analysis are additionally shown. Channels: blue: Hoechst, red: propidium iodide, green: caspase 3; Annotations (A-F): cyan border: edge of the cerebellar slice, yellow border surrounding detections visually classified to be propidium iodide positive nuclei / Caspase 3 positive cells, red border surrounding detections visually classified to be ignored; Detections (D – F): yellow detections: detections classified as propidium positive nuclei / Caspase 3 positive cells by the object classifier, red detections: detections classified by the object classifier to be ignored.

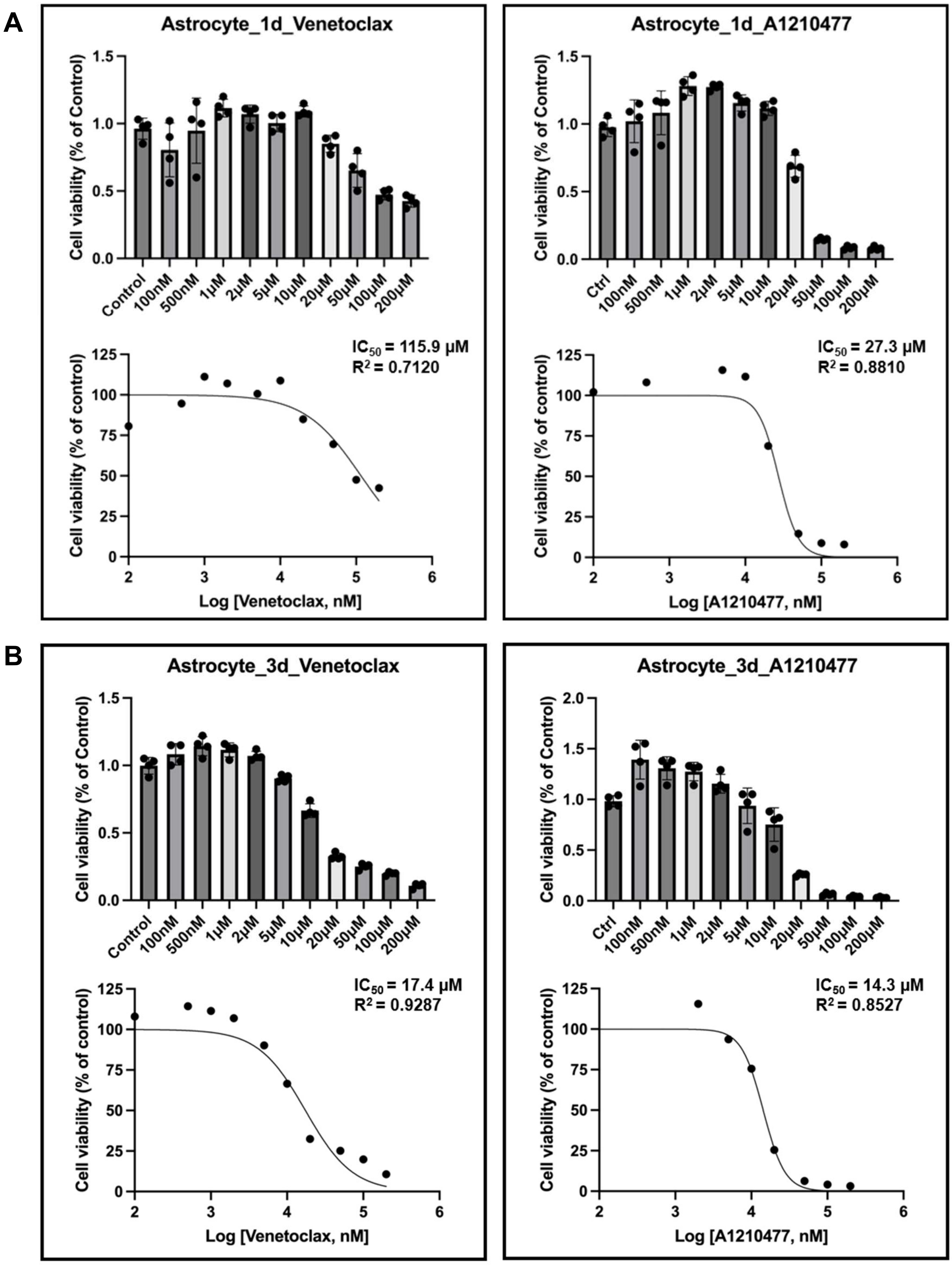

Figure S2. Cell viability curves for Venetoclax and A1210477 tested in primary astrocytes after 1 (**A**) and 3 days (**B**), respectively. IC50 values for both drugs were determined and depicted with their corresponding R^2-values. All data are from experiments performed in quadruplicates; data are presented as mean ± SD.

Table S1. IC_{50} values for Venetoclax and A1210477 for GB cell lines U87 and U251. All concentrations were determined by triplicates. Number of viable cells were determined by CellTiter-Glo reagent and IC_{50} values were calculated using non-linear regression with the least-square fit.

Cells	IC_{50} (nM)			
	1d		3d	
	**Venetoclax**	**A1210477**	**Venetoclax**	**A1210477**
**U87**	32.6×10^3	15.2×10^3	7.7×10^3	7.8×10^3
**U251**	129.8×10^3	19.5×10^3	18.6×10^3	8.9×10^3
